# The Epigenetic Regulation of Microenvironment in Hepatocellular Carcinoma

**DOI:** 10.3389/fonc.2021.653037

**Published:** 2021-03-15

**Authors:** Fang Wang, Greg Malnassy, Wei Qiu

**Affiliations:** ^1^Department of Surgery, Loyola University Chicago Stritch School of Medicine, Maywood, IL, United States; ^2^Department of Cancer Biology, Loyola University Chicago Stritch School of Medicine, Maywood, IL, United States

**Keywords:** tumor microenvironment, DNA methylation, histone methyltransferases, histone deacetylases, microRNAs, hepatic stellate cells, tumor-associated macrophages, extracellular matrix

## Abstract

Hepatocellular carcinoma (HCC) is a highly lethal and complex malignancy strongly influenced by the surrounding tumor microenvironment. The HCC microenvironment comprises hepatic stellate cells (HSCs), tumor-associated macrophages (TAMs), stromal and endothelial cells, and the underlying extracellular matrix (ECM). Emerging evidence demonstrates that epigenetic regulation plays a crucial role in altering numerous components of the HCC tumor microenvironment. In this review, we summarize the current understanding of the mechanisms of epigenetic regulation of the microenvironment in HCC. We review recent studies demonstrating how specific epigenetic mechanisms (DNA methylation, histone regulation, and non-coding RNAs mediated regulation) in HSCs, TAMs, and ECM, and how they contribute to HCC development, so as to gain new insights into the treatment of HCC via regulating epigenetic regulation in the tumor microenvironment.

## Introduction

Hepatocellular carcinoma (HCC) is the fourth most common cause of cancer death worldwide (1, 2). HCC patients' prognosis is low, with a 5-year survival of just 18% (2). For many years, the only FDA-approved systemic treatment option for advanced HCC was the multi-kinase inhibitor sorafenib, which itself provided only a modest increase in overall survival of 2.8 months compared to placebo (3). More recently, other therapeutics have garnered first-line treatment approval, including lenvatinib and atezolizumab combined with bevacizumab (4, 5). Second-line treatment options for relapsed and refractory HCC have also expanded in recent years to include regorafenib, cabozantinib, and the PD-1 checkpoint inhibitors nivolumab and pembrolizumab (6–9). While the arsenal of therapies for advanced HCC has expanded, all aforementioned agents are marked by low response rates and limited improvements in overall survival (4–9). Understanding the molecular mechanisms underlying HCC development are critical to identifying new drug candidates capable of providing enhanced clinical benefit.

In recent years, researchers have started to decipher the complicated crosstalk between the tumor and the surrounding tumor microenvironment (TME). The TME is composed of numerous different elements depending on the tumor type but generally includes surrounding vasculature, immune cells, fibroblasts, and the underlying extracellular matrix (ECM). TME has been repeatedly shown to significantly contribute to tumor initiation, progression, invasiveness, metastases formation, and angiogenesis (10–12). In the context of HCC, the TME primarily consists of hepatic stellate cells (HSCs), tumor-associated macrophages (TAMs), the ECM, mesenchymal stem/stromal cells (MSCs), myeloid-derived suppressor cells (MDSCs), and endothelial cells (ECs) with each element performing its unique role in HCC pathogenesis (13–21).

Epigenetics is the heritable modification of gene function without changes in the DNA sequence, which is mediated by a number of different factors, including but not limited to, DNA methylation, histone modifications, and non-coding RNAs (22, 23). Epigenetic regulation in tumor cells is extensively studied and plays critical roles in HCC development (24–26). Emerging evidence demonstrates that epigenetic alterations in TME also contribute to the initiation and progression of HCC (27–33). As the epigenetic regulation of MSCs, MDSCs, and ECs in HCC remain poorly defined, this review will focus on the epigenetics of HSCs, TAMs, and the ECM (Table 1).

**Table 1:** The epigenetic regulation of tumor microenvironment in hepatocellular carcinoma.

	**Hepatic stellate cells (HSCs)**	**Tumor-associated macrophages (TAMs)**	**Extracellular matrix (ECM)**
DNA methylation	• DNMT1-mediated hypermethylation of *PTEN, H19*, and *RCAN1.4* promotes HSC activation. • DNMT3a-mediated DNA methylation of *PTGIS* and *Septin9* promotes HSC activation. • DNMT3b-mediated hypermethylation of *SUN2* promotes HSC activation. • Increased expression of MAT2A and MAT2β results in global DNA hypomethylation and promotes HSC activation. • NNMT induced by HSCs activation promotes HCC metastasis.	Methylation of CSF1R regulates TAM trafficking and promotes HCC growth.	Suppressed expression of TIMP3 by promoter methylation promotes HCC development.
Histone modifications	• P300 HAT promotes HSC activation and HCC metastasis. • HDAC3 promotes HSC activation by regulating TGFβ expression. • HDAC4 promotes HSC activation by regulating MMP9 and MMP13. • MLL1 promotes ethanol-induced HSC activation. • EZH2 promotes HSC activation by downregulating PPARγ. • JMJD2D promotes HSC activation by regulating TLR4 transcription. • SIRT6 deacetylates *SMAD3* and suppresses HSC activation. • HDAC7 suppresses HSC activation by inhibiting HGF expression. • JMJD1A inhibits HSC activation by regulating PPARγ.	• SIRT1 facilitates M1 macrophage polarization and suppresses HCC metastasis. • SIRT4 inhibits macrophage activation and HCC growth.	• HDAC4 represses the expression of MMP9 and MMP13 in HCC cells. • EZH2 decreases MMP9 expression in HSCs.
Non-coding RNAs	MiRNAs that promote HSC activation: • MiR-21, MiR-221, MiR-151, MiR-214, MiR-542-3p, MiR-942, MiR-146b, MiR-17-5p, MiR-34a, MiR-125a-5p MiRNAs that suppress HSC activation: • MiR-96-5p, MiR-23b/27b, MiR-338-3p, MiR-378, MiR-155, MiR-146a-5p, MiR-9-5p, MiR-130-3p, MiR-30a	• MiR-98 targets IL10 to modulate macrophage polarization, thus stifling the effects of TAMs. • MiR-101 targets DUSP1 to inhibit TAM-induced HCC growth. • Decreased miR-28-5p targets IL-34 to suppress TAM infiltration and HCC growth. • Reduced miR-125a and miR-125b and increased miR-15b secret from TAMs promote HCC growth. • lncRNA cox-2 reduces HCC by suppressing M2 macrophage polarization • LncRNA H19, induced by TAMs, promotes HCC progression.	MiRNAs that downregulated MMPs and TIMPs expression: • MMP2: MiR-29b; MiR-107 • MMP9: MiR-107; MiR-328-3p; MiR-133a • TIMP2: MiR-519d • TIMP3: MiR-191; MiR-181b; MiR-221/222

### Hepatic Stellate Cells

HSCs are resident perisinusoidal cells that contribute to diverse aspects of liver physiology, including hepatic organogenesis, regeneration, vitamin A storage, and wound healing (34). Under non-pathologic conditions, HSCs remain quiescent in the liver and are only activated in response to liver injury (35). In addition to serving as the primary source of ECM proteins, activated HSCs secrete a multitude of cytokines and growth factors that are required for fibrogenesis and promote HCC tumorigenesis (34–39). Namely, HSCs can modulate the ECM through secretion and upregulation of matrix metalloproteinases (MMPs), such as MMP2 and MMP9, both of which promote HCC tumor migration (40–43). Moreover, activated HSCs promote FAK-MMP9 signaling and invasiveness in HCC, highlighting the crosstalk between tumor cells and act ivated HSCs in the hepatic TME (44–46). The activation of HSCs is the result of extensive, but reversible, alterations in gene expression: activated HSCs can reclaim their quiescent state upon regression/resolution of liver damage in a dynamic process that is modulated extensively by epigenetic reprogramming (38, 47–49).

#### DNA Methylation in HSC Activation

DNA methylation influences HSC activation and activity. Activation of HSCs *in vitro* results in significant changes in DNA methylation and treatment with the DNA methylation inhibitor 5-aza-2'-deoxycytidine (5-azadC) block can block HSC transdifferentiation (50, 51). DNA methyltransferase 1 (DNMT1)-mediated hypermethylation of the Phosphatase and tensin homolog (PTEN) promoter leads to a loss of PTEN expression, subsequent activation of the PI3K/AKT and ERK pathways, and HSC activation (52). DNMT1 expression is increased in activated HSCs and rat liver fibrosis tissue, which leads to enhanced methylation of the lncRNA H19 promoter and elevates H19 expression and ERK activation. Treatment with 5'-aza-2'-deoxycytidine in activated HSCs model reduced fibrosis-associated gene expression as well as DNMT1 expression while simultaneously enhancing H19 expression and attenuated HSCs activation. Consistently, sennoside A can prevent liver fibrosis by binding DNMT1 and suppress DNMT1-mediated PTEN hypermethylation in HSC activation and proliferation (53).

Inhibition of DNA methyltransferase 3a (DNMT3a) causes activated HSCs to lose the fibrogenic phenotype in a carbon tetrachloride (CCl_4_)-induced liver fibrosis model (54). In addition to PTEN, DNA methylation of prostacyclin synthase (PTGIS) enhances HSC activation and liver fibrogenesis, with its methylation being induced by DNMT1 as well as DNMT3b, as determined by chromatin immunoprecipitation (ChIP) (55). DNMT3a has also been reported to regulate the methylation of Septin9 to promote hepatic stellate cell activation and liver fibrogenesis (56).

SAD1/UNC84 domain protein-2 (SUN2) gene hypermethylation at CpG sites has also been identified during liver fibrogenesis in mice with CCl_4_-induced hepatic fibrosis, accompanied by low expression of SUN2 (57). *In vivo* overexpression of SUN2 following adeno-associated virus-9 (AAV9) administration inhibited CCl_4_-induced liver injury and reduced fibrogenesis marker expression. Mechanistically, DNMT3b is the principal regulator of SUN2 expression, and inhibition of AKT phosphorylation may be a crucial pathway for SUN2-mediated HSC activation. S-adenosylmethionine suppresses the expression of Smad3/4 in activated human hepatic stellate cells via Rac1 promoter methylation (58). Methylation of RCAN1.4 is mediated by DNMT1 and DNMT3b and enhances HSC activation and liver fibrogenesis through Calcineurin/NFAT3 signaling (59).

Methionine adenosyltransferases (MATs) catalyze the biosynthesis of S-adenosylmethionine (SAMe), the principal methyl donor in DNA methylation. Methionine adenosyltransferase 2A and 2B (MAT2A and MAT2B), the sole regulators of the SAMe homeostasis in HSCs, are induced during *in vitro* and *in vivo* HSC activation. MATII enzyme activity and intracellular SAMe levels decline during HSC activation, accompanied by a global decrease in DNA methylation. MAT2A and MAT2B are induced during HSC activation and are essential for this process. The SAMe level falls, resulting in global DNA hypomethylation (60). Elevated nicotinamide N-methyltransferase (NNMT) expression induced by hepatic stellate cells promotes HCC metastasis by altering the histone H3 methylation and transcriptionally activating CD44 (61).

#### Histone Modification in HSC Activation

Histone modification also plays a critical in HSC activation. Acetylation and methylation are the two most extensively characterized histone modifications. Histone acetylation and methylation are carried out by two families of enzymes: histone acetyltransferases (HATs) and histone methyltransferases (HMTs). In opposition to these enzymes, histones are deacetylated and demethylated by histone deacetylases (HDACs) and histone demethylases (HDMTs), respectively. Both epigenetic marks can change quickly in response to intracellular and environmental cues and confer immense control of cellular responses (62). The p300 HAT is involved in stiffness-mediated HSC activation to promote liver tumor metastasis (63). Mechanistically, stiffness induces p300 nuclear accumulation, which, in turn, promotes the p300-dependent transcription of critical fibrogenic genes and several secreted profibrogenic and tumor-promoting factors. Furthermore, Sirtuin 6 (SIRT6), a NAD-dependent histone deacetylase which is a critical epigenetic regulator in alcoholic liver disease (64), deacetylates Smad family member 3 (Smad3) and attenuates its expression induced by transforming growth factor β (TGF-β) in the activated HSCs (65).

HSC activation and transdifferentiation are accompanied by gene expression changes in numerous HDACs. Notably, HDAC1, HDAC2, HDAC9, and HDAC10 were reported to be downregulated during HSC activation, while the expression of HDAC4, HDAC5, HDAC6, and HDAC8 is increased (66). MC1568, a class II selective HDAC4 and HDAC5 inhibitor, abrogates HSC activation and proliferation *in vitro* and *in vivo* (67). Inhibition of HDAC1, HDAC2, and HDAC4 by nilotinib is also known to induce HSC cell death (68). The mechanisms by which several of these HDACs regulate HSC activation and activity have been characterized in detail. For example, HDAC4 accumulates during HSC activation, and forced overexpression of HDAC4 in quiescent HSCs suppresses expression of the endopeptidases MMP9 and MMP13 resulting in ECM accumulation (66, 67, 69). Conversely, genetic knockdown of HDAC4 in activated HSCs promotes the expression of MMP9 and MMP13, thereby promoting ECM degradation. HDAC7 is also known to contribute to HSC activation by regulating the expression of hepatocyte growth factor (HGF), inhibiting HSC activation and liver fibrosis (70). The expression of HGF is reduced during the activation of HSCs, and knockdown of HDAC7 restores HGF levels to quiescent HSC levels (70). HDAC3 is required to activate HSCs by regulating TGF-β, which plays a crucial role in ECM formation and remodeling (71). Consistently, HDAC inhibitors such as SAHA suppress HSC activation and liver fibrosis by attenuating the TGF-β signaling pathway (72).

Besides HDACs, HMTs regulate gene expression during HSC activation. Ethanol exposure promotes HSC activation by inducing the expression of mixed lineage leukemia protein-1 (MLL1), a histone 3 lysine 4 (H3K4) methyltransferase. Following increased expression, MLL1 is recruited to the elastin gene promoter, where it is associated with increased H3K4me3 levels, increased ECM deposition, and HSC transdifferentiation (73). During HSC activation, several histone methyltransferases, such as ASH2, WDR5, and SET1, are recruited to the promoters of pro-fibrogenic genes in response to TGF-β treatment by myocardin-related transcription factor A (MRTF-A) (74). Enhancer of zeste homolog 2 (EZH2), the catalytic methyltransferase subunit of the polycomb repressive complex 2 (PCR2), is another HMT that regulates HSC activation and hepatic fibrosis. EZH2 expression is induced in HSCs upon TGF-β treatment in both *in vitro* and CCl_4_-treated mouse livers (75). Upregulation of EZH2 downregulates expression of peroxisome proliferator-activated receptor-gamma (PPARγ), a nuclear receptor essential for HSC activation (76, 77).

Histone demethylation by HDMTs is also a process critical to HSC activation. Jumonji domain-containing protein 1A (JMJD1A) is one of these HDMTs. JMJD1A knockdown in HSCs increases H3K9me2 levels on the PPARγ gene promoter and represses the expression of PPARγ (78). JMJD2D, a histone H3 demethylase, also contributes to HSC activation by demethylating H3K9 residues. The result of this demethylation is increased TLR4 transcription and activation of the TLR4/MyD88/NF-kB signaling pathway, which has a well-established role in liver fibrosis (79). JMJD2D expression is markedly increased in activated HSCs, and AAV9 shRNA-mediated knockdown of JMJD2D suppresses hepatic fibrosis in the CCl_4_ model of liver fibrosis (80).

#### Micro-RNA Involvement in HSC Activation

miRNAs are small, non-coding RNAs (20–25 nucleotides in length) that regulate gene expression by binding to target mRNA transcripts and subsequently facilitating the transcript's cleavage, destabilization, or inhibiting its translation (81). MiRNAs can also control the activation of HSCs by several different mechanisms (38). Namely, HCC cells secrete extracellular vesicles (EV) with oncogenic miRNAs (oncomiRs), specifically miR-21, miR-221, and miR-151, which can activate HSCs. This activation promotes HCC cell invasion, epithelial to mesenchymal transition (EMT), and activation of the AKT/ERK signaling pathway (82). miR-214 promotes HSC activation by inhibiting the expression of suppressor-of-fused homolog (Sufu), a negative regulator of Hedgehog signaling pathway (83). miR-542-3p is also capable of stimulating HSC activation and fibrosis by targeting bone morphogenetic protein 7 (BMP7), a potent surpressor of TGF-β signaling (84). miR-942, which is induced by both TGF-β and LPS, assists HSC activation through downregulation of BMP and activin membrane-bound inhibitor homolog (BAMBI) (85). miR-146b boosts HSC activation by targeting of Kruppel-like factor 4 (KLF4) (86). miR-17-5p targets Smad7 thus stimulating the activation of HSCs (87). Likewise, miR-34a facilitates HSC activation through decreasing the expression of acyl-CoA synthetase-1 (ACSL1) (88). miR-125a-5p advances activation of HSCs by targeting a negative regulator of hypoxia-inducible factor 1 (HIF1), the aptly named factor inhibiting HIF-1 or FIH1 (89).

Conversely, several miRNAs have been shown to suppress HSC activation. miR-96-5p suppresses the activation of HSCs by inhibiting ATG7 and autophagy (90). The miR-23b/27b cluster bind to 3′-UTR of gremlin 1, contributing to the reduction of TGF-β expression and HSC inactivation (91). Additionally, miR-338-3p inhibits HSC activation and proliferation through targeting cyclin-dependent kinase 4 (CDK4) (92). mi-378 represses HSC activation by targeting Gli3 and reducing the expression of Gli3 (93). miR-155 inhibits STAT5 and FOXO3a expression, thus silencing HSC activation (94). miR-146a-5p abrogates HSC activation by inhibiting TGF-β1/Smad and LPS/NF-κB signaling pathways (95, 96). Similarly, both miR-9-5p and miR-130-3p attenuate the activation of HSCs by targeting TGFβR1 and TGFβR2 (97). miR-30a impedes HSC activation through the inhibition of EMT via directly targeting SNAI1 (98).

### Tumor-Associated Macrophages

Macrophages are essential innate immune cells in the tumor microenvironment and are also closely associated with cancer occurrence, metastases, and disease progression (99). Tumor-associated macrophages (TAMs) that reside in the surrounding TME are crucial components in the HCC immune microenvironment for the formation and development of HCC (100, 101). TAMs promote HCC growth, angiogenesis, invasion, and migration while simultaneously suppressing the antitumor immune response (20). The epigenetic regulation of TAMs has been shown to be critical to their differentiation and functional programming (102–105).

#### DNA Methylation and Histone Modification in TAMs

DNA methylation and histone modification are critical regulatory mechanisms in TAMs for promoting tumor growth and progression (106–109). However, the regulation of DNA methylation and histone modification of TAMs in HCC remains poorly understood. A recent study showed that colony-stimulating factor 1 receptor (CSF1R), which plays an essential role in forming TAMs (108, 110), is regulated by DNA methylation in HCC (111). DNA methylation leads to decreased expression of CSF1R in HCC tissues and associates with poor clinicopathological characteristics of HCC (111). SIRT1, a NAD-dependent histone deacetylase, suppresses HCC metastasis by facilitating M1 macrophage polarization via the stimulation of the NF-κB pathway (112). Downregulation of SIRT4, another sirtuin protein, in TAMs promotes macrophages activation and HCC growth via the FAO-PPARδ-STAT3 axis (113).

#### miRNAs and Long Non-coding RNAs in TAMs

The epigenetic remodeling by miRNA regulates macrophages' activation and functional programming (114–117). For example, the down-regulation of miR-98 was accompanied by up-regulation of IL-10 in TAMs of HCC (118). miR-98 modulates macrophage polarization from M2 to M1 in HCC by targeting IL-10, thus stifling the effects of TAMs on advancing EMT and HCC metastasis (118, 119). miR-101 targets dual-specificity phosphatase 1 (DUSP1) to inhibit TAM-induced HCC growth through TGF-β secretion regulation (120). Decreased expression of miR-28-5p suppresses HCC growth and metastasis by directly targeting interleukin-34 (IL-34) and affecting IL-34-mediated TAM infiltration (121). Reduced expression of miR-125a and miR-125b secreted from TAMs promote HCC proliferation and cancer stem cell properties directly targeting CD90 (122). miR-15b secreted from TAMs promotes the aggressiveness of HCC by impeding the LATS1-mediated hippo pathway (123).

Long non-coding RNAs (lncRNAs) are a class of RNAs >200 nucleotides in length, which lack protein-coding capabilities and have gained increased attention in recent years due to their ability to function guides, signals, and decoys in a highly tissue-specific manner (124). Several lncRNAs have been shown to participate in tumorigenesis and cancer progression, a number of which do so by modulating elements of the TME (125, 126). In the context of HCC, lncRNA cox-2 reduces HCC immune evasion and metastasis formation by suppressing M2 macrophage polarization (127). lncRNA H19, induced by TAMs, promotes the progression of HCC via regulating the miR-193b/MAPK1 axis (128).

### Epigenetics in the Extracellular Matrix

The ECM plays a critical role in physiological and pathological processes in HCC (129). MMPs, which digest the ECM, are key players in ECM remodeling and are associated with tumor growth and invasion through collagen and matrix degradation (130, 131). Metallopeptidase inhibitor proteins (TIMPs) are natural inhibitors of MMPs (132). Epigenetic modification is a critical mechanism for regulating MMP and TIMP expression, thus playing a pivotal role in HCC development (133).

#### DNA Methylation and Histone Modification in MMPs and TIMPs

Overexpression of MMPs, such as MMP2 and MMP9, is frequently observed in HCC patients and associated with cancer invasive potential (41, 42). While some studies have shown that DNA methylation can regulate the expression of MMPs in other cancers, it remains unknown if DNA methylation directly regulates the expression of MMPs in HCC (134). Emerging evidence, however, indicates that DNA methylation promotes the dysregulation of TIMPs in HCC. Specifically, TIMP3 suppresses HCC cell proliferation and survival, and TIMP3 expression is suppressed by promoter methylation in HCC cells (135, 136). Histone deacetylation by HDAC4 represses MMP promoter activities and the expression of MMP9 and MMP13 in HSCs (66, 137). Sodium butyrate, a histone deacetylase inhibitor, decreases MMP1 mRNA expression in human liver cancer cells (138). Further, overexpression of histone methyltransferase EZH2 decreases MMP9 expression in HSCs (139).

#### miRNAs in MMPs and TIMPs

miRNAs are also involved in the Regulation of MMP and TIMP expression (140). For instance, miR-29b directly targets MMP2 and suppresses HCC growth (141). The blocking of MMP2, either by neutralizing antibody or RNA interference, phenocopies the anti-invasion and antiangiogenesis effects of miR-29b, whereas, the introduction of MMP2 antagonizes the function of miR-29b in HCC (141). miR-107 has also been shown to downregulate MMP2 and MMP9 in HCC cells (142). miR-328-3p and miR-133a modulate the expression of MMP9 to inhibit HCC cell proliferation (143, 144). miR-191 promotes EMT and HCC tumor growth by directly targeting TIMP3 and inhibiting TIMP3 expression (145). MiR-181b and miR-221/222 can also target TIMP3 to promote HCC growth (146, 147). Moreover, miR-519d targets TIMP2 and promotes HCC cell proliferation and metastasis (148).

## Conclusion and Future Perspectives

**Figure 1:**
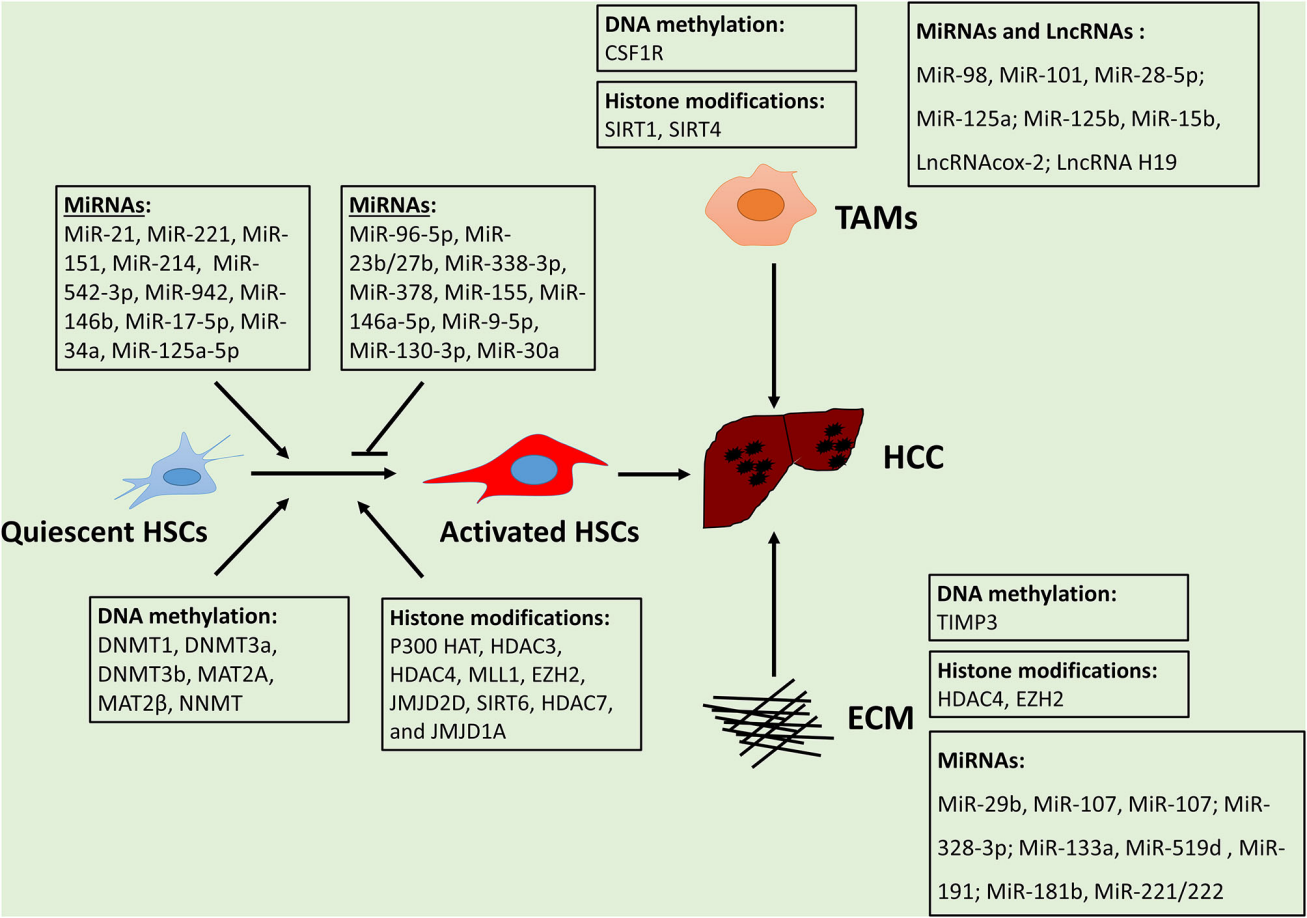
HCC microenvironment components and their epigenetic regulation.

The tumor microenvironment plays a critical role in HCC initiation and progression. Epigenetic regulation is a crucial mechanism for the alteration of TMEs in HCC. Overall, epigenetic reprogramming extensively modulates gene expression alterations in HSCs, TAMs, and ECMs, thus promoting HCC development (Figure 1). The effect of metabolic factors (e.g., SAMe) on epigenetic alterations of TMEs in HCC is evident, suggesting dysregulation metabolites establish can affect the epigenome and subsequently impact HCC development. The mechanisms of epigenetic regulation in MSCs, MDSCs, and ECs remain poorly investigated, thus necessitating future study. Understanding the interactions between tumors and TMEs will further enhance our understanding of critical drivers or suppressors for tumor progression. Despite substantial advances in understanding the mechanisms of TMEs, developing therapeutics using this knowledge remains a challenge. Further examination and deciphering of the complex epigenetic landscape of the TME in HCC will help identify new targets and strategies for the treatment of HCC.

## Author Contributions

All authors listed have made a substantial, direct and intellectual contribution to the work, and approved it for publication.

## Conflict of Interest

The authors declare that the research was conducted in the absence of any commercial or financial relationships that could be construed as a potential conflict of interest.
